# There is detectable variation in the lipidomic profile between stable and progressive patients with idiopathic pulmonary fibrosis (IPF)

**DOI:** 10.1186/s12931-021-01682-3

**Published:** 2021-04-09

**Authors:** Shabarinath Nambiar, Britt Clynick, Bong S. How, Adam King, E. Haydn Walters, Nicole S. Goh, Tamera J. Corte, Robert Trengove, Dino Tan, Yuben Moodley

**Affiliations:** 1 Separation Science and Metabolomics Laboratory, Murdoch University, Murdoch, WA Australia; 2 School of Biomedical Science, University of Western Australia, Crawley, WA Australia; 3 Institute for Respiratory Health, Nedlands, WA Australia; 4 Metabolomics Australia, Murdoch University, Murdoch, WA Australia; 5 Scientific Operations, Waters Corporation, Stamford Avenue, Wilmslow, SK9 4AX UK; 6 Alfred Hospital, Melbourne, VIC Australia; 7 University of Tasmania, Hobart, TAS Australia; 8 University of Melbourne, Parkville, VIC Australia; 9 Royal Hobart Hospital, Hobart, TAS Australia; 10 Austin Hospital, Heidelberg, VIC Australia; 11 Institute of Breathing and Sleep, Heidelberg, VIC Australia; 12 University of Sydney, Camperdown, NSW Australia; 13 Royal Prince Alfred Hospital, Camperdown, NSW Australia; 14 Fiona Stanley Hospital, Murdoch, WA Australia

**Keywords:** Lipids, Plasma, IPF, MS, DIA, SONAR

## Abstract

**Background:**

Idiopathic pulmonary fibrosis (IPF) is a chronic interstitial lung disease characterized by fibrosis and progressive loss of lung function. The pathophysiological pathways involved in IPF are not well understood. Abnormal lipid metabolism has been described in various other chronic lung diseases including asthma and chronic obstructive pulmonary disease (COPD). However, its potential role in IPF pathogenesis remains unclear.

**Methods:**

In this study, we used ultra-performance liquid chromatography-quadrupole time-of-flight mass spectrometry (UPLC-QTOF-MS) to characterize lipid changes in plasma derived from IPF patients with stable and progressive disease. We further applied a data-independent acquisition (DIA) technique called SONAR, to improve the specificity of lipid identification.

**Results:**

Statistical modelling showed variable discrimination between the stable and progressive subjects, revealing differences in the detection of triglycerides (TG) and phosphatidylcholines (PC) between progressors and stable IPF groups, which was further confirmed by mass spectrometry imaging (MSI) in IPF tissue.

**Conclusion:**

This is the first study to characterise lipid metabolism between stable and progressive IPF, with results suggesting disparities in the circulating lipidome with disease progression.

## Background

Idiopathic pulmonary fibrosis (IPF) is a chronic, progressive lung disease characterized by alveolar epithelial cell activation and damage, resulting in proliferation of activated fibroblasts and extracellular matrix deposition leading to irreversible destruction of gas exchange units and lung remodelling [1]. Previous studies suggested that IPF is related to abnormalities in a number of biological processes including glycolysis, fatty acid oxidation and vascular remodelling [1–3]. Lipidomics and metabolomics are a developing field of systems biology research that studies lipids as key intermediates of cellular mechanisms and their roles have been explored in other respiratory diseases [4, 5].

Ultra-performance liquid chromatography-quadrupole time-of-flight mass spectrometry (UPLC-QTOF-MS) is a powerful tool for qualitative characterization of chemical components, providing acquisition of MS spectra with relatively high resolution, sensitivity and mass accuracy, which has become an advanced tool for the qualitative and quantitative analysis of multiple components. Lipidomic studies of complex biological matrices (plasma and tissue extracts) are routinely performed using UPLC-QTOF-MS, resulting in excellent metabolite detection performance at high speeds and sensitivities [6]. A key challenge in the analysis of complex lipid extracts from crude plasma samples is the co-elution of isomeric lipid species as a result of small variations in their fatty acid arrangements and hydrocarbon backbones [7]. To help overcome this, we have applied SONAR, a rapidly scanning method in tandem with UPLC-QTOF-MS acquisition to provide additional information and quantification of metabolites in complex samples [8, 9].

There have been several studies examining circulatory molecules that characterise progressive IPF, focusing mostly on proteomics and genomics [10–12]. Two studies have characterised lipidome differences between IPF and healthy controls, reporting several lipids that have the ability to differentiate IPF from controls [13, 14]. We therefore hypothesize that there are differences in circulating lipids between stable and progressive IPF. To address this, we carried out UPLC-QTOF-MS implementing SONAR, to assess plasma samples from stable and progressive IPF patients.

## Methods

### Biological samples

The study cohort consisted of a total of 58 plasma samples (30 stable and 28 progressors) from the Australian IPF Registry with a clinical diagnosis of IPF (Table 1). Baseline forced vital capacity (FVC) and diffusing capacity for carbon monoxide (DLco) were assessed ± 6 months from the time of blood collection, and the longitudinal FVC and DLco trajectories were determined ± 6–12 months from the baseline lung function using a linear regression model. A decline in FVC ≥ 10% and/or DLco ≥ 15% within 6–12 months of baseline was used to define progressive IPF. All work was approved by the Sydney Local Health District Human Research Ethics Committee (Reference number: HREC 11/RPAH 439), Royal Perth Hospital Human Research Ethics Committee (Reference number: REG 15-204) and the Murdoch University Human Research Ethics Committee (Approval number: 2017/254).

**Table 1:** Summary of the clinical characteristics of stable versus progressive IPF patients

Characteristic	Number of cases	
	Stable	Progressive
All cases	30	28
Age (years)		
Mean	68 ± 8	70 ± 9
Sex		
Male	19	17
Female	11	11
Smoking history		
Never	13	8
Ex-smoker	16	20
Current	1	–
FVC (% predicted)	82 ± 20	73 ± 15
DLCO (% predicted)	53 ± 18	37 ± 14

Age, FVC and DLCO displayed as mean values (± SD)

*SD* standard deviation, *FVC* forced vital capacity, *DLCO* diffusing capacity for carbon monoxide

### Sample preparation

Plasma lipid extraction was carried out as previously described [15], and each sample was transferred into two different plates corresponding to positive and negative modes of acquisition. This allows ions to be differentiated by its charge (protinate for positively charged and deprotinated for negatively charged molecules). This is useful where the stability of the molecule being characterised is affected by its charge and which mode it runs through, which may also provide complementary structural information through different fragmentation processes [16]. Pooled samples were generated and used as the study reference or for quality control (QC) checks.

### UPLC configuration

Ultra-performance liquid chromatography (UPLC) is an analytical technique used for the separation, identification and further quantitation of compounds in a mixture using pressurized organic and/or polar solvent systems. This was used for the separation, identification and further quantitation of compounds using an ACQUITY I-class system (Waters Corporation, USA) equipped with a Waters CSH C18 column (2.1 × 100 mm, 1.8 µm). Chromatographic separation was achieved with a gradient of 40 to 99% mobile phase over 18 min. Solvent flow rate and column temperature was maintained at 0.4 mL/min and 55 °C, respectively. The lock-mass compound, leucine enkaphlin (200 pg/μL) was prepared in acetonitrile/H_2_O (50:50, v/v), and was delivered at 10 µL/min to the reference sprayer source of the mass spectrometer.

### Mass spectrometry acquisition

MS-based lipid analysis was performed using a Xevo G2-XS QTOF mass spectrometer (Waters Corporation, UK) in positive electrospray ionisation (ESI) mode. Note, positive ionization investigates positive ions in low pH, and negative ionization investigates negative ions at high pH. The source temperature and capillary voltage was set to 120 °C and 2.0 kV, respectively. The time-of-flight (TOF) mass analyser of the mass spectrometer was calibrated using a mass to size ratio (m/z) 50 to 1200.

### Data processing

Peak picked features were statistically analysed using EZinfo (MKS Data Analytics Solutions, Sweden) and the significant features of interest were imported back into Progenesis QI. Lipid identification was achieved by matching experimental fragments against the theoretical fragmentation product ion spectra from LIPID MAPS structure database (Lipidomics Gateway, UK). Additional manual annotation was also conducted using the Lipid Reporter toolkit and reported according to the shorthand nomenclature defined by Liebisch et al*.* [17].

### Statistical analysis

The coefficient of variation (CV) was calculated across quality control (QC) samples for each feature and those with a CV > 30% were removed. The high CVs at these levels demonstrate the expected loss of precision when quantifying samples at the extreme ends of the assay's range. Two statistical modelling tools were used to discriminate diseased experimental groups, including the principal component analysis (PCA) and orthogonal projection to latent structures-discriminant analysis (OPLS-DA), based on their contribution to the variation and correlation between the two groups (stable versus progressors). These modeling tools provide insights into separations between experimental groups based on the collected high-dimensional spectral measurements. PCA was initially performed to deconstruct the stable and progressive patient dataset; however, the unsupervised multivariate approach did not reveal significant differences between both groups (Fig. 2a). The heterogeneity between these two diseased groups with similar underlying mechanisms were more likely to cluster together in a principal component space. Supervised multivariate data analysis OPLS-DA was then used to generate a regression model to disentangle group-predictive and group-unrelated variation in the measured data. The OPLS-DA model clearly distinguished the stable and progressor groups from which an S-plot was then generated (Fig. 2b, c). The S-Plot is a statistical tool for visualizing both the covariance and correlation between the endogenous features and the modelled group designation (stable versus progressor). Consolidation of the S-Plots from the OPLS-DA models were useful for the identification of biochemically atypical features with statistical significance between the groups, based on their contributions to the model and their reliabilities. Furthermore, variable importance in projection (VIP) scores of each feature were calculated from the OPLS-DA model, summarising the contributions each lipid makes to the model. Features with VIP scores ≥ 1 were considered as significant [18].

### MALDI-MSI acquisition

In order to validate whether changes in the circulation is present in the lung, mass spectrometry imaging (MSI) was carried out to spatially resolve the distribution of the biomolecules resolved by SONAR. The lipids were resolved in a total of 20 fresh frozen tissue sections (20 µm; 10 from IPF patients and 10 healthy controls) using matrix-assisted laser desorption ionisation (MALDI)-QTOF MSI and data was interrogated by High Definition Imaging (HDI) software (Waters Corporation, U.K.) to generate ion intensity maps. The heat maps of a specific ion generated corresponded to the relative abundance of ions present over the entire imaged surface. All data were acquired in positive mode operating over a mass range of m/z 50 to 1200 and were performed using the Water Synapt G2S mass spectrometer equipped with an orthogonal MALDI ion source and an Nd:YAG laser (Waters Corporation, Manchester, U.K.). Putative identification of lipids was achieved by accurate mass measurement and matched against lipid databases including LIPID MAPS and LipidBlast.

## Results

Demographic characteristics of this Australian cohort (Table 1) demonstrated for the stable cohort predominantly males (n = 19, 63%), mean age (68 ± 8 year), FVC 82 ± 20% predicted and DLco 53 ± 18% predicted; and for the progressive cohort predominantly males (n = 17, 61%), mean age (70 ± 9 year), FVC 73 ± 15% predicted and DLco 37 ± 14% predicted.

**Fig. 1:**
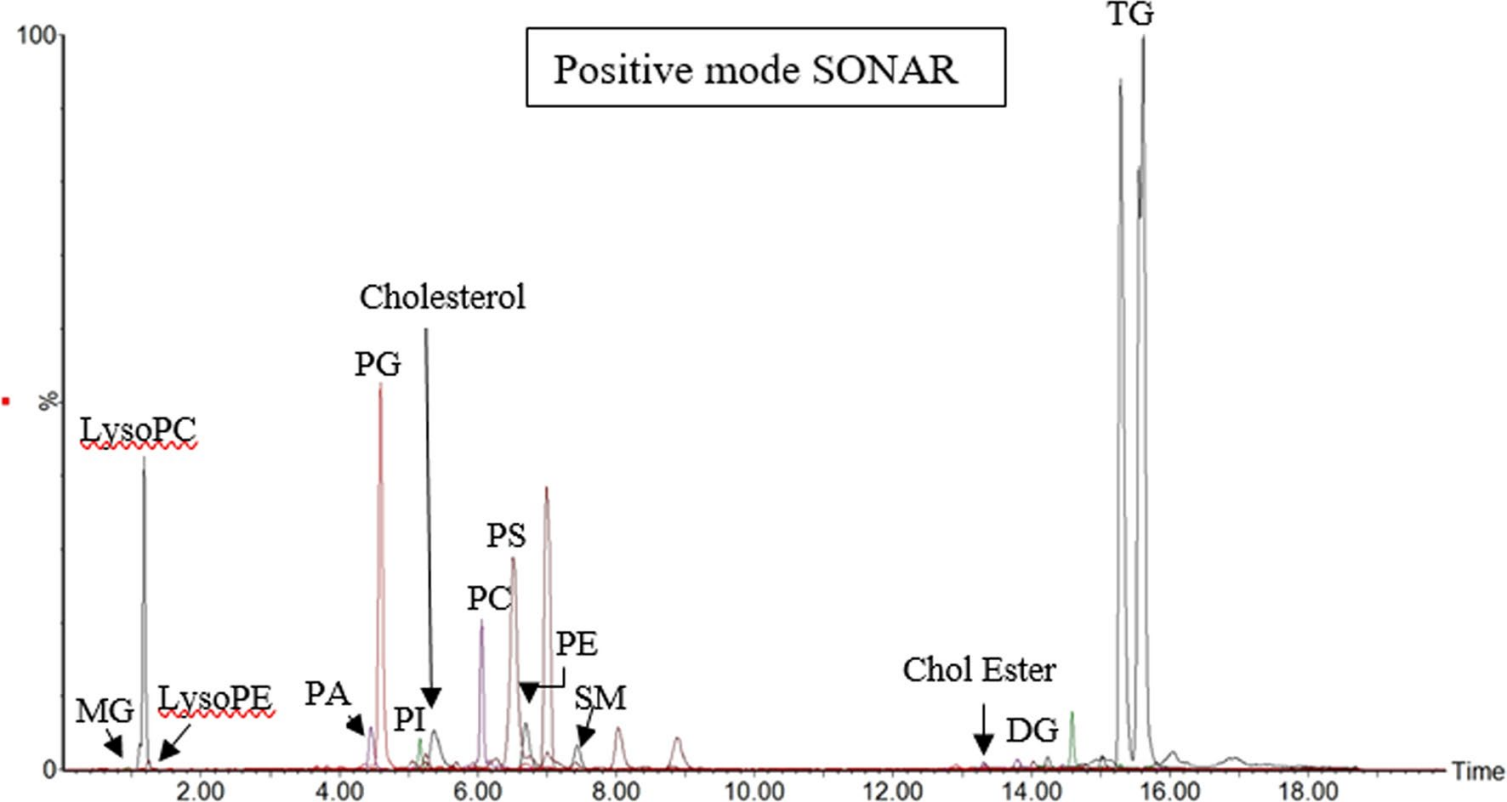
Total ion chromatograms for SONAR acquisitions in positive mode. The extracted ion chromatograms of spiked deuterium-labelled SPLASH LipidoMix standards in positive ion mode showing peaks corresponding to 15:0–18:1(d7) phosphatidylcholine (PC), 15:0–18:1(d7) phosphatidylethanolamine (PE), 15:0–18:1(d7) phosphatidylserine (PS), 15:0–18:1(d7) phosphatidylglycerol (PG), 15:0–18:1(d7) phosphatidylinositol (PI), 15:0–18:1(d7) phosphatidic acid (PA), 18:1(d7) lysophosphatidylcholine (LysoPC), 18:1(d7) lysophosphatidylethanolamine (LysoPE), 18:1(d7) cholesteryl ester (Chol Ester), 18:1(d7) monoglyceride (MG), 15:0–18:1(d7) diacylglycerol (DG), 15:0–18:1(d7)-15:0 triglyceride (TG), 18:1(d9) sphingomyelin (SM) and cholesterol (d7)

With SONAR, approximately 5000 features were resolved in each analytical run. The total ion chromatograms (TIC) also displayed a typical lipid spectral pattern consistent with previous studies [19]. All lipid classes were resolved by SONAR (Fig. 1).

**Fig. 2:**
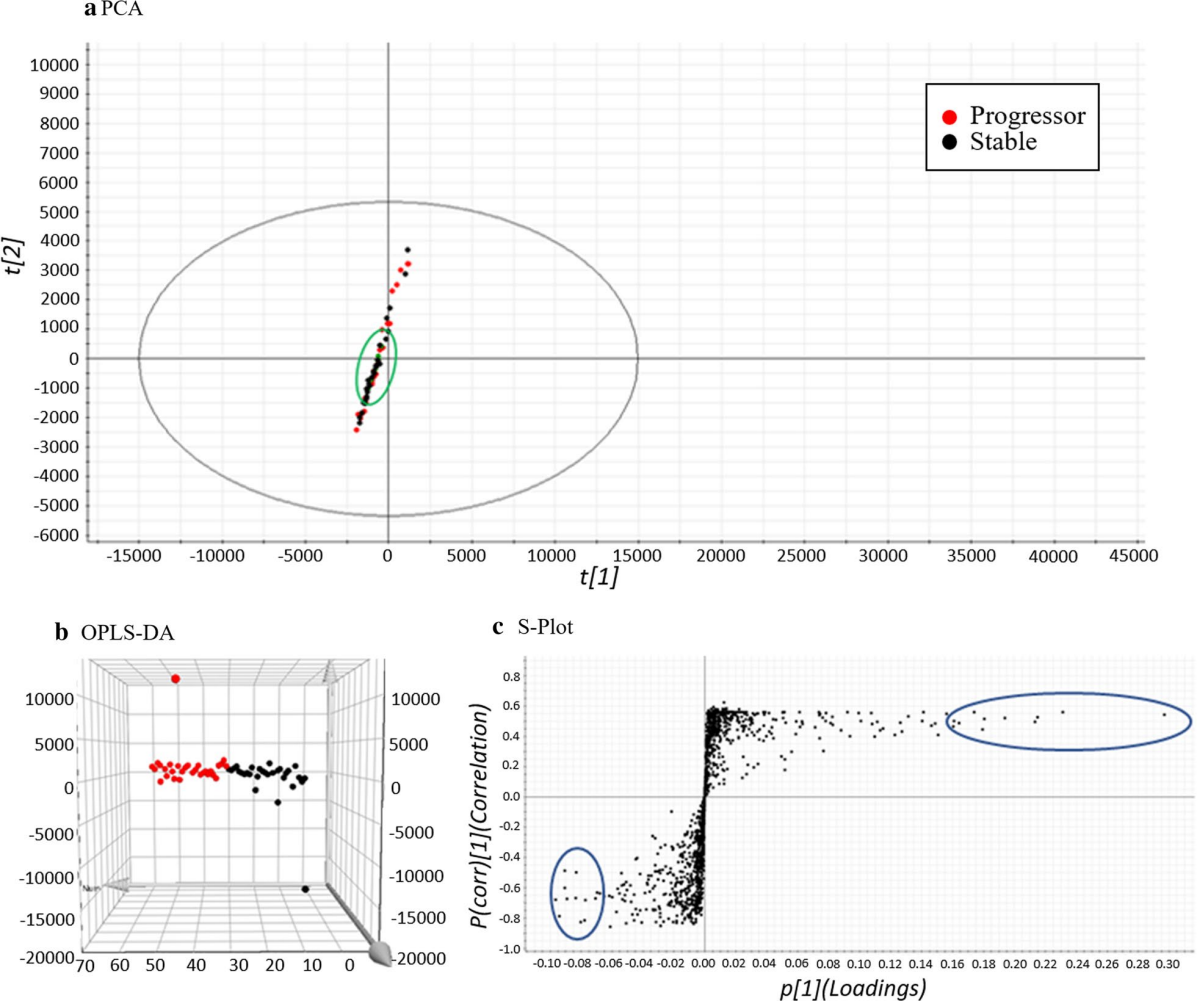
Statistical modelling used to discriminate diseased experimental groups. **a** PCA score plots generated from all stable (black) and progressor (red) and QC (green) samples in both modes of acquisition. The clustering of the pooled QC samples in each acquisition modes were shown encircled in green. **b** The OPLS-DA and **c** S-plots show comparisons between stable (black) versus progressors (red) plasma samples in the aforementioned acquisition modes. The ten features of interest in each group (encircled in blue) were exported into Progenesis QI software for identification

For statistical analysis, PCA was initially performed to decompress the stable and progressive patient dataset; however, unsupervised multivariate analysis did not reveal significant differences between the groups (Fig. 2a). Supervised multivariate data analysis OPLS-DA was then used to generate a regression model to disentangle group-predictive and group-unrelated variation in the measured data [20]. The OPLS-DA model clearly distinguished the stable and progressor groups from which an S-plot was then generated (Fig. 2b, c) [21].

From the features exported using EZinfo, eight lipids from positive SONAR mode were putatively identified. The identified lipids included six glycerolipids (triglycerides (TG)) and two glycerophoslipids (phosphatidylcholine (PC)) (Table 2). Using SONAR, three TGs (54:5, 54:6, 53:7) and two PCs (40:6, 36:3) were resolved higher in the progressor subgroup (between 1.9–3.3-fold change). All other lipids observed relatively unchanged levels between the two subgroups (between 0.9–1.11-fold differences).

**Table 2:** Plasma lipids identified between stable and progressive IPF

MS mode	Feature *m/z*	Putative ID	Fold change
Positive SONAR	878.8190	TG [52:1]	1.02
	898.7870	TG [54:5]	1.85
	896.7712	TG [54:6]	3.33
	904.8352	TG [54:2]	1.05
	822.7554	TG [48:1]	0.90
	880.7468	TG [53:7]	2.32
	834.6008	PC [40:6]	1.94
	784.5847	PC [36:3]	2.18

*MS* mass spectrometry, *TG* triglyceride, *PC* phosphatidylcholine

**Fig. 3:**
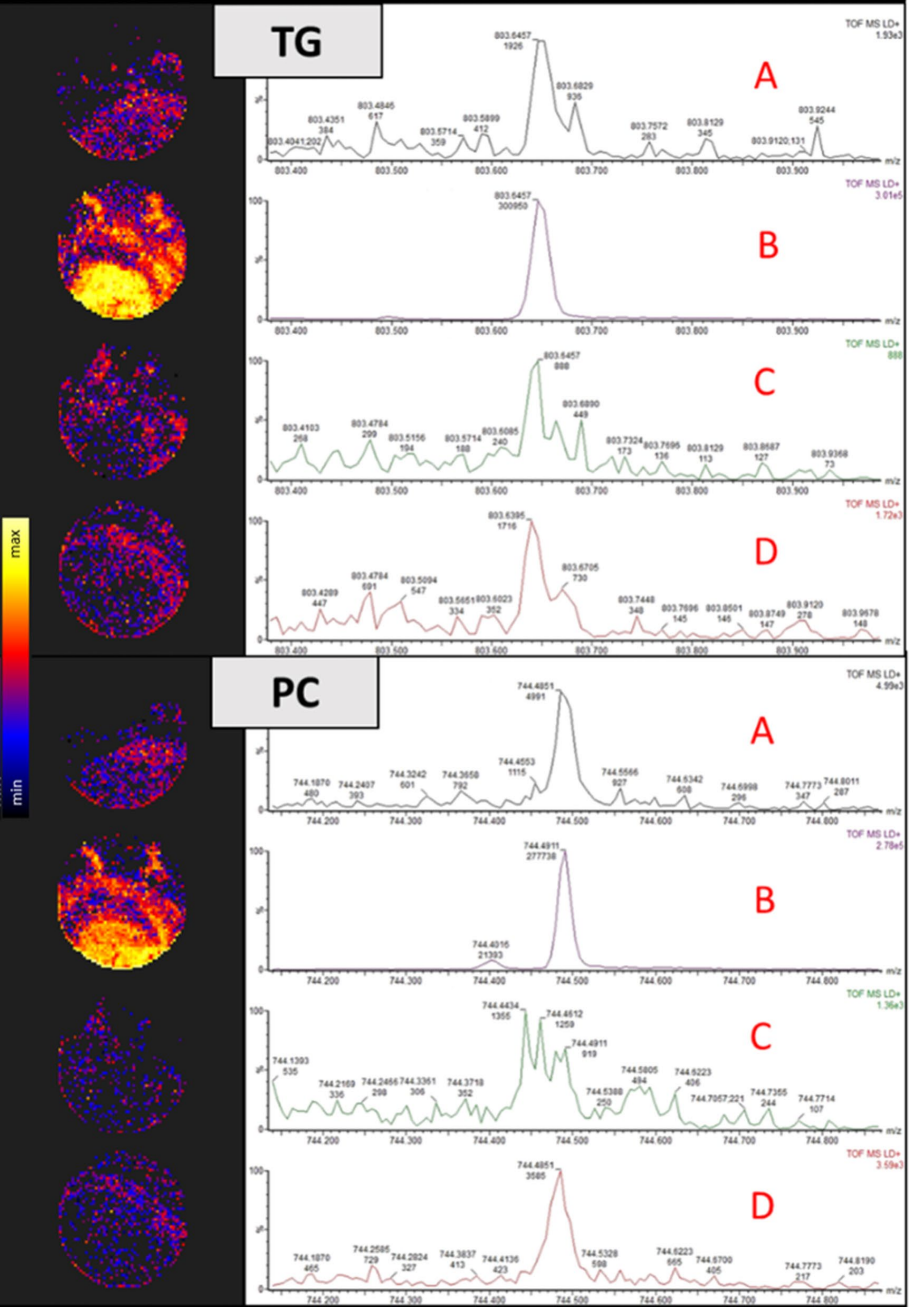
Extracted ion chromatograms and ion intensity maps of the lipid adducts generated by Mass Lynx and HD Imaging software, respectively. phosphatidylcholine (PC) and triglycerides (TG) were extracted and their associated adducts [M+NH_4_]^+^, [M+K]^+^, [M+Na]^+^ and [M+H]^+^ are denoted by A, B, C and D, respectively

Exploration of these lipids in IPF tissue revealed an abundance of PC and TG, corresponding to circulatory findings. More specifically, the adduct species [M+K]^+^ showed improved abundance, while [M+H]^+^ and [M+NH_4_]^+^ showed poor ion spectra (Fig. 3). In this study, these enhanced signal intensities was advantageous as it allowed for the improved discrimination of lipid analytes from the background ions. This is backed up by previous research indicating that [M+K]^+^ ions were the only adduct type that were well-resolved for the two lipids [22, 23].

## Discussion

Lipids play an important role in lung pathology and physiology. Although the composition and involvement of the lipidome in various diseases is still poorly understood, abnormal lipid metabolism has been reported in few lung diseases including asthma and COPD [4, 5]. Lipids comprise diverse classes of molecules which are critically involved in cellular structure, signalling and energy storage [24]. However, their potential role in IPF pathogenesis remains unclear. This is the first study to evaluate the differences in the lipidome of stable versus progressive IPF patients.

Another novel aspect of this study was the tandem use of SONAR in assessing differences in plasma samples between stable and progressive IPF. It is well documented that conventional quadrupole time-of-flight (QTOF)-MS methods are successful at resolving the lipid classes chromatographically, however, compound identification is still challenging due to the inaccurate assignment of lipid precursors to their corresponding product ions. In contrast, the precursor and product ions generated by SONAR contributed to the specificity of the method, increasing the probability of successful lipid library matching [8, 25]. Furthermore, the MALDI-MSI method allowed for the visualisation of these lipids within IPF tissue.

With SONAR, a number of TG and PC were identified and found to be expressed at higher levels in the IPF progressor group. Specifically, the levels of these TGs (53.7, 54:5 and 54:6) and PCs (36:3 and 40:6) appeared to be elevated in samples of progressors compared to stable patients. This trend is consistent with the findings of Yan et al. [13] and Kulkarni et al. [14] and although their observations were based on comparisons between IPF and healthy controls, higher levels in progressive IPF could relate to more active disease process. Specifically, using a bleomycin (BLM) mouse model of pulmonary fibrosis, Kulkarni et al. [14] observed unchanged levels of specific TGs (48:1, 52:1 and 54:2) consistent with our results, but reported a marked increase of approximately twofold in the relative content of PCs. This suggests that these species may play a role in the pathogenesis of BLM-induced pulmonary fibrosis.

TGs along with diglycerides (DGs), are the most abundant lipids found in circulating plasma. Serum total TG has been reported as a biomarker of fatty acid metabolic disturbance [26]. TGs are stored in lipid droplet structures, formed through budding of the endoplasmic reticulum (ER), and are reported to induce the expression of endogenous ER stress markers (e.g. p-JNK, GRP 78) [27, 28]. Interestingly, ER stress is evident in alveolar epithelium of both human and mice with pulmonary fibrosis [29]. However, neither the mechanisms causing this stress, nor its contribution to fibrosis is well understood. Further investigation of the exact roles of TGs in IPF patients would be beneficial.

Unlike TGs, the functional role of PCs has been described in lung surfactant phospholipid metabolism. Pulmonary surfactant forms the lining of the epithelial air-contact surface of the lungs and are essential for the prevention of alveolar collapse during expiration. PC is the major phospholipid comprising around 80% of surfactant lipids and alterations in their composition can cause reduced elasticity, leading to an overall decrease in lung compliance [30]. Changes in the component phospholipids, including PCs have been described in bronchoalveolar fluids of animal models of rapidly developing pulmonary fibrosis [31]. The up-regulated PC in this study might be partially associated with greater epithelial injury in the progressive group relative to stable.

To understand the global dynamics of metabolite differences in IPF, we performed a metabolic network analysis. We found four networks enriched in the progressive IPF samples (Linoleic acid metabolism, alpha-Linolenic acid metabolism, Arachidonic acid metabolism, Glycerophospholipid metabolism), which have been previously described in fibrotic lung [32, 33]. Based on these findings, it has been proposed that there is a fine balance between lipid metabolism and wound healing mechanisms leading to fibrosis onset and development in IPF [32].

Although a limitation in this study is its small cohort size and the lack of a healthy cohort, abnormalities in plasma lipids in IPF versus healthy controls have been previously described [13], 14] forming the basis of our lipidomic exploration. The biological significance pertaining to the fold change differences will need to be further explored in larger cohorts and with functional studies.

## Conclusion

In conclusion, this project successfully profiled plasma samples obtained from two groups of IPF patients using the SONAR acquisition approach enhancing the specificity of unbiased lipid profiling derived from UPLC-QTOF-MS. In particular, we have identified changes related to disease progression in lipid signatures such as TGs and PCs in IPF plasma samples as whole, which have previously been associated with abnormalities in lipid metabolism through mitochondrial-beta oxidation pathways.

## Data Availability

All data generated or analysed during this study are included in this published article (and its additional information files).

## Abbreviations

COPD: Chronic obstructive pulmonary disease
CV: Coefficient of variation
DIA: Data-independent acquisition
DLCO: Diffusing capacity for carbon monoxide
ER: Endoplasmic reticulum
ESI: Electrospray ionisation
FVC: Forced vital capacity
HDI: High definition imaging
IPF: Idiopathic pulmonary fibrosis
m/z: Mass to size ratio
MALDI: Matrix-assisted laser desorption ionisation
MS: Mass spectrometry
MSI: Mass spectrometry imaging
OPLS-DA: Orthogonal projections to latent structures discriminant analysis
PC: Phosphatidylcholines
PCA: Principal component analysis
QC: Quality control
QTOF: Quadrupole time-of-flight
TG: Triglycerides
TIC: Total ion chromatograms
TOF: Time-of-flight
UPLC-QTOF-MS: Ultra-performance liquid chromatography-quadrupole time-of-flight mass spectrometry
VIP: Variable importance in projection
